# Prevalence and associated factors of erectile dysfunction, psychological disorders, and sexual performance in primary vs. secondary infertility men

**DOI:** 10.1186/s12958-021-00720-5

**Published:** 2021-03-09

**Authors:** Jianxiong Ma, Yingying Zhang, Binghao Bao, Wangqiang Chen, Haisong Li, Bin Wang

**Affiliations:** 1 The Second Clinical Medical College, Zhejiang Chinese Medical University, Hangzhou, China; 2 Department of Andrology, Dongzhimen Hospital, Beijing University of Chinese Medicine, Beijing, China; 3 Key Laboratory of Integrative Chinese and Western Medicine for Prevention and Treatment of Sexual Dysfunction of Zhejiang Province, Hangzhou, China; 4 Department of Reproductive Medicine, Zhejiang Provincial Integrated Chinese and Western Medicine Hospital, Hangzhou, China

**Keywords:** Infertility, Erectile dysfunction, Depression, Anxiety, Risk factors

## Abstract

**Background:**

Many infertile couples might experience erectile dysfunction (ED) and significant changes in the quality of sexual life and psychological state though information is limited in secondary infertile men in China. To determine whether primary or secondary infertility is associated with ED, psychological disorders, and sexual performance.

**Methods:**

This was a cross-sectional survey conducted at the Dongzhimen Hospital of Beijing University of Chinese Medicine (06/2019-01/2020). The participants completed a questionnaire including general information, sexual life, simplified International Index of Erectile Function (IIEF-5), Patient Health Questionnaire-9 (PHQ-9), and 7-item Generalized Anxiety Disorder Scale (GAD-7). Multivariable logistic regression was used to identify the factors associated with ED, depression, and anxiety.

**Results:**

ED was more frequent in secondary vs. primary infertility (46.5 % vs. 26.7 %, *P* < 0.001). Compared with men with primary infertility, those with secondary infertility showed lower IIEF-5 scores (*P* < 0.001), higher occurrence of TOIF (*P* = 0.001), had a higher awareness of partner’s ovulation when having ED (*P* = 0.001), lower GAD-7 scores (*P* = 0.016), lower libido (*P* = 0.005), fewer intercourses per month (*P* = 0.001) and a lower sexual satisfaction score (*P* = 0.027). In the multivariate analysis, primary infertility was found to be an independent risk factor of anxiety (OR: 1.812, 95 %CI: 1.015–3.236). Some overlap is observed in factors associated with ED, psychological disorders, and sexual performance between primary and secondary infertility, but some factors are distinct.

**Conclusions:**

The prevalence of ED in secondary infertility men was higher than that of primary infertility men, and the quality of sexual life was decreased. Primary infertility is an independent risk factor of anxiety.

## Introduction

Infertility is the inability to conceive after 1 year of unprotected sexual intercourse [1–3]. Infertility occurs in about 15 % of reproductive-aged couples worldwide and is more common in developing countries [1]. Most researchers and clinicians habitually refer to those without pregnancy as primary infertility, while those with infertility after a first pregnancy or for 12 months after stopping contraceptives as secondary infertility [4].

In 2016, the family planning policy changed from a one-child policy to a two-child policy in China, which rekindles the need for births in many families. Still, many couples who want to have a second child may be beyond the appropriate age and be with low fertility, resulting in a large increase in the number of patients with secondary infertility.

Many infertile couples will experience multiple problems, especially significant changes in the quality of sexual life and psychological state [5–7]. Previous studies showed that the incidence of erectile dysfunction (ED) in men with infertility is higher than that in the general population [8–10]. Infertile men also often experience depression and anxiety due to self-inflicted, couple, and social pressure [11, 12]. The negative impacts of depression and anxiety on couples’ sexual life, marriage happiness, and quality of life are self-evident. In different studies, the reported prevalence of depression and anxiety in infertile men ranged from 4.9 to 38 % [13–16]. In addition, there is an interaction between ED and psychological condition, suggesting that these aspects have to be monitored in infertile couples [17, 18]. Nevertheless, there is limited information on the incidence and related risk factors of ED, depression, and anxiety in secondary infertile men in China. It might be beneficial to investigate this population and obtain relevant clinical data to assist clinicians in improving the treatment plans for infertile couples.

Therefore, this study aimed to determine whether primary or secondary infertility is associated with ED, psychological disorders, and sexual performance and to investigate whether the associated factor of ED, psychological disorders, and sexual performance are different between men with primary and secondary infertility. The results might help clinicians make better decisions when managing infertility.

## Methods

### Study design and participants

This was a cross-sectional survey conducted at the andrology clinic of Dongzhimen Hospital of Beijing University of Chinese Medicine from June 2019 to January 2020. The study was approved by the medical ethics committee of Dongzhimen Hospital of Beijing University of Chinese Medicine. Written informed consent was obtained from each participant.

The inclusion criteria were: (1) the male partner of a married couple with pregnancy plans; (2) settled down and living with his wife; (3) regular intercourse with wife and without contraception for at least one year [19]; (4) sought medical help because his wife could not conceive; and (5) agreed to participate in the survey and signed the informed consent form. The exclusion criteria were: (1) obvious genital malformation; (2) severe cardiovascular or cerebrovascular disease; (3) liver, kidney, or mental disease; or (4) hypertension, diabetes mellitus, or chronic obstructive pulmonary disease.

### Questionnaires

The participants were surveyed using an anonymous questionnaire that included general information (age, height, weight, education level, marriage duration, and couple’s relationship), fertility assessment (primary or secondary infertility, cause of infertility, and history of semen collection for assisted reproductive technology), International Index of Erectile Function (IIEF-5), sexual life (awareness of partner’s ovulation period, timely ovulatory intercourse failure [TOIF], self-reported sexual desire, intercourse frequency, intra-vaginal ejaculation latency time [IELT], and sexual satisfaction), Patient Health Questionnaire-9 (PHQ-9), and 7-item Generalized Anxiety Disorder Scale (GAD-7).

Each participant filled out a paper questionnaire and recorded their sexual activity six months before the survey. It took about 5–10 min to complete the questionnaire. Andrology physicians or medical interns with uniform training guided the participants to complete the questionnaire independently and explained the items to those with reading or understanding problems.

The IIEF-5 was used to assess the occurrence and severity of erectile dysfunction (ED). The IIEF-5 score ranges from 5 to 25, with 22–25 indicating no ED, 12–21 indicating mild ED, 8–11 indicating moderate ED, and 5–7 indicating severe ED [20]. The Chinese version of IIEF-5 has been validated [21]. TOIF was defined as the inability to have an erection or maintaining an erection for satisfactory ejaculation during intercourse with the female partner during the ovulation period. Sexual satisfaction was assessed using a Likert-7 scale, with 1 to 7 reflecting very dissatisfied to very satisfied. The PHQ-9 [22] was used for the screening of depression and the assessment of symptom severity. It consists of nine items, each scored 0 to 3. PHQ-9 scores of 0–4 indicated no depressive symptom, 5–9 indicated mild depressive state, and ≥ 10 indicated confirmed depression, and its sensitivity and specificity are above 90 % [23]. The PHQ-9 has been validated in Chinese [24]. GAD-7 [25] was used for the screening of generalized anxiety and the assessment of symptom severity. It consists of seven items, each scored 0 to 3. The GAD-7 score was 0–4 for no anxiety symptom, 5–7 for mild anxiety state, ≥ 8 for confirmed anxiety, and its sensitivity and specificity were above 90 % [26]. The GAD-7 has been validated in Chinese [27].

### Statistical analysis

Data were analyzed using SPSS 21.0 (IBM, Armonk, NY, USA). Continuous variables were expressed as means ± standard deviations. Continuous data with a normal distribution were analyzed using Student’s t-test, while data with a skewed distribution were analyzed using the Mann-Whitney U-test. Categorical variables were expressed as frequencies (percentages) and analyzed using the chi-square test. Variables with *P* < 0.20 in the univariable logistic regression analyses were included in the multivariable stepwise logistic regression analysis to analyze the independent factors associated with ED, depression, and anxiety. The odds ratios (ORs) and 95 % confidence intervals (CIs) were calculated. *P*-values < 0.05 were considered statistically significant.

## Results

### Characteristics of the participants

This study enrolled 387 married men who met the study inclusion criteria. The participants were divided into the primary (*n* = 258) and secondary (*n* = 129) infertility groups according to whether they already had children. The patient characteristics are summarized in Table 1. Compared with the patients who had primary infertility, those with secondary infertility were older (38.8 ± 5.7 vs. 31.4 ± 3.8 years, *P* < 0.001), had a higher body mass index (BMI) (24.9 ± 3.4 vs. 24.3 ± 3.1 kg/m^2^, *P* = 0.05), had a higher monthly income (*P* = 0.001), had a lower education level (*P* = 0.003), had a longer marriage (6.8 ± 5.8 vs. 3.2 ± 2.1 years, *P* < 0.001), and showed a higher proportion of infertility caused by both male and female factors (*P* < 0.001).

**Table 1:** Characteristics of the patients

Characteristics	Total (*n* = 387)	Primary infertility (*n* = 258)	Secondary infertility (*n* = 129)	*P*
Age (years), mean ± SD	33.9 ± 5.7	31.4 ± 3.8	38.8 ± 5.7	< 0.001
BMI (kg/m^2^), mean ± SD	24.5 ± 3.2	24.3 ± 3.1	24.9 ± 3.4	0.05
Monthly income (RMB), n (%)				0.001
0-5000	10 (2.6)	9 (3.5)	1 (0.8)	
5001-10,000	44 (11.4)	30 (11.6)	14 (10.9)	
10,001–15,000	114 (29.5)	85 (32.9)	29 (22.5)	
15,001–20,000	131 (33.9)	89 (34.5)	42 (32.6)	
> 20,000	88 (22.7)	45 (17.4)	43 (33.3)	
Education level, n (%)				0.003
Junior high school and lower	10 (2.6)	3 (1.2)	7 (5.4)	
High school	62 (16.0)	32 (12.4)	30 (23.3)	
University	228 (58.9)	160 (62.0)	68 (52.7)	
Above university	87 (22.5)	63 (24.4 %)	24 (18.6)	
Marriage duration (years), mean ± SD	4.4 ± 4.1	3.2 ± 2.1	6.8 ± 5.8	< 0.001
Couple’s relationship, n (%)				0.194
Average	40 (10.3)	23 (8.9)	17 (13.2)	
Good	347 (89.7)	235 (91.1)	112 (86.8)	
ART semen collection, n (%)				0.743
No	286 (73.9)	192 (74.4)	94 (72.9)	
Yes	10 1(26.1)	66 (25.6)	35 (27.1)	
Cause of infertility, n (%)				< 0.001
Male factors	181 (46.8)	128 (49.6)	53 (41.1)	
Female factors	47 (12.1)	33 (12.8)	14 (10.9)	
Both factors	100 (25.8)	49 (19.0)	51 (39.5)	
Unknown	59 (15.2)	48 (18.6)	11 (8.5)	

*SD* standard deviation, *BMI* body mass index, *ART* assisted reproduction technology

### Erectile dysfunction, psychological disorders, and quality of sexual life among infertile men 

Table 2 shows that there are significant differences in ED (including prevalence, IIEF-5 score and TOIF), GAD score and sexual life (including sexual life satisfaction score and frequency) between primary and secondary infertile men.

**Table 2:** Erectile dysfunction, psychological disorders, and quality of sexual life among infertile men

	Total (*n* = 387)	Primary infertility (*n* = 258)	Secondary infertility (*n* = 129)	*P*
**Erectile dysfunction**				
Severity of ED, n (%)				< 0.001
Without ED	258 (66.7)	189 (73.3)	69 (53.5)	
Mild	115 (29.7)	65 (25.2)	50 (38.8)	
Moderate	11 (2.8)	3 (1.2)	8 (6.2)	
Severe	3 (0.8)	1 (0.4)	2 (1.6)	
IIEF-5 score, mean ± SD	21.2±3.9	21.7 ± 3.5	20.0 ± 4.6	< 0.001
TOIF, n (%)				0.001
Never appeared	270 (69.8)	191 (79.6)	79 (62.7)	
< 50 %	70 (18.1)	38 (15.8)	32 (25.4)	
≥ 50 %	26 (6.7)	11 (4.6)	15 (11.9)	
Partner ovulation, n (%)				0.570
Known	366 (94.6)	240 (93.0)	126 (97.7)	
Unknown	21 (5.4)	18 (7.0)	3 (2.3)	
People who know partner’s ovulation, n (%)				0.001
Without ED	270 (74.0)	191 (79.6)	79 (62.7)	
With ED	95 (26.0)	38 (15.8)	32 (25.4)	
**Psychological disorder**				
PHQ-9 score, mean ± SD	5.5 ± 4.1	5.5 ± 4.2	5.5 ± 3.9	0.685
Severity of depression, n (%)				0.621
None	185 (47.8)	127 (49.2)	58 (45.0)	
Mild	144 (37.2)	91 (35.3)	53 (41.1)	
Confirmed diagnosis	58 (15.0)	40 (15.5)	18 (14.0)	
GAD-7 score, mean ± SD	6.2 ± 3.8	6.8 ± 4.2	6.2 ± 3.8	0.016
Severity of anxiety, n (%)				0.075
None	152 (39.3)	96 (37.2)	56 (43.4)	
Mild	100 (25.8)	63 (24.4)	37 (28.7)	
Confirmed diagnosis	135 (34.9)	99 (38.4)	36 (27.9)	
**Sexual life**				
Sexual desire, n (%)				0.005
Normal	229 (59.1)	167 (65.0)	62 (48.4)	
General	110 (28.4)	66 (25.7)	44 (34.4)	
Low	46 (38.7)	24 (9.3)	22 (17.2)	
Intercourse frequency per month, mean ± SD	5.3 ± 2.1	5.6 ± 2.1	4.8 ± 2.0	0.001
IELT (min), mean ± SD	5.9 ± 3.6	6.0 ± 3.5	5.7 ± 3.6	0.299
Sexual satisfaction score, mean ± SD	5 (4–6)	6 (4–6)	5 (3–6)	0.027

*SD* standard deviation, *ED* erectile dysfunction, *IIEF-5* simplified International Index of Erectile Function, *TOIF* timely ovulation intercourse failure, *IELT* intra-vaginal ejaculation latency time, *PHQ-9* Patient Health Questionnaire-9, *GAD-7* 7-item Generalized Anxiety Disorder Scale

Indeed, compared with men with primary infertility, those with secondary infertility showed a higher frequency of ED (46.5 % vs. 26.7 %, *P* < 0.001). This difference is supported by a lower IIEF-5 scores in men with secondary infertility (20.0 ± 4.6 vs. 21.7 ± 3.5, *P* < 0.001). In addition, a higher occurrence of TOIF was found in the secondary fertile group (37.3 % vs. 20.4 %, *P* = 0.001). Table 2 also shows that the severity of anxiety and depression and the PHQ-9 score were not significantly different between the two groups, but the GAD-7 scores was lower in the secondary infertility group (*P* = 0.016), suggesting that anxiety symptoms were more severe in the secondary infertility group than in the primary infertility group. Regarding sexual life, the men with secondary fertility had lower libido (*P* = 0.005), fewer intercourses per month (*P* = 0.001), and a lower sexual satisfaction score (*P* = 0.027) than the men with primary infertility.

**Fig. 1:**
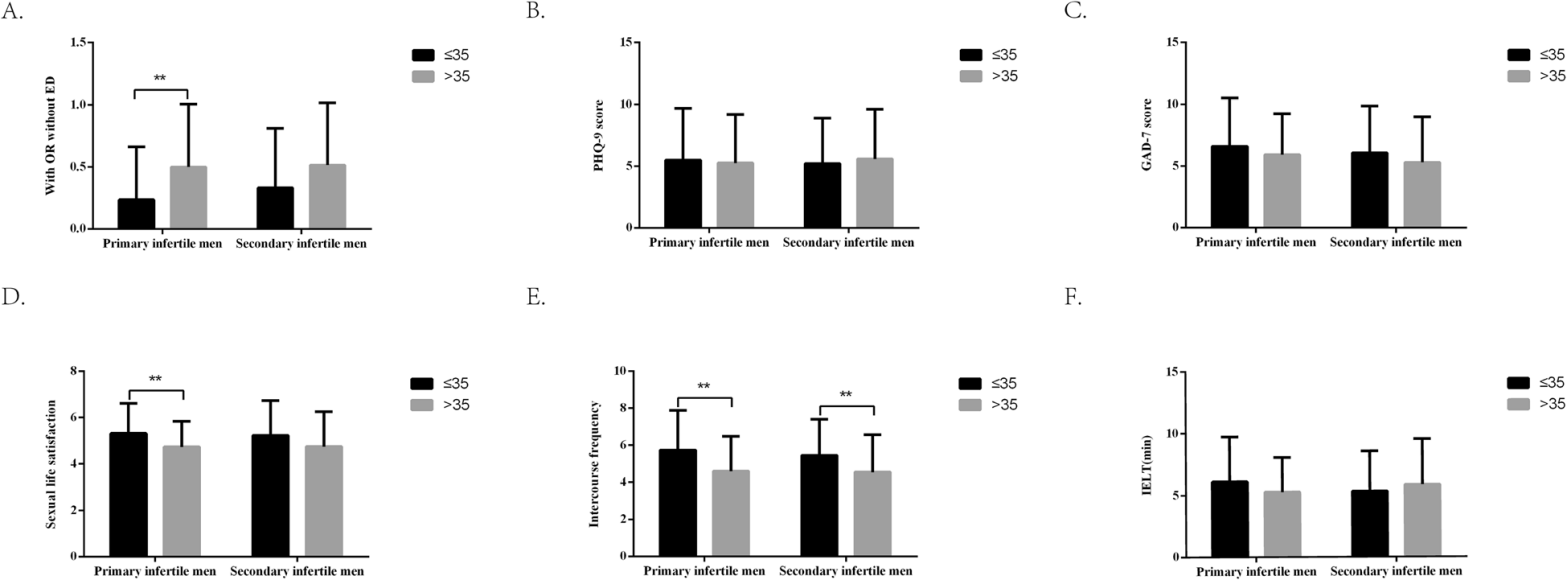
Erectile dysfunction, psychological disorders, and quality of sexual life by age among patients with different infertile type. **a** Prevalence of erectile dysfunction among patients with different infertility type and by age. Comparisons of age groups, by infertility type, for (**b**) PHQ-9, **c** GAD-7 score, **d** sexual satisfaction, **e** intercourse frequency, and **f** intra-vaginal ejaculation latency time (IELT). ** represents *P* < 0.05

Figure 1 shows that the prevalence of ED and sexual life satisfaction score were significantly different by age in primary infertile men but not secondary infertile men. Indeed, the prevalence of ED was higher among men with primary infertility > 35 years of age compared with those ≤ 35 years of age (*P* = 0.002). The sexual satisfaction was lower among men with primary infertility > 35 years of age compared with those ≤ 35 years of age (*P* = 0.020). Intercourse frequency was lower in men > 35 years of age compared with those < 35 years of age, both in primary (*P* = 0.006) and secondary (*P* = 0.024) infertility.

### Multivariable logistic regression analysis of ED, depression, and anxiety risk factors in all patients

Table 3 shows that primary infertility is an independent risk factor of anxiety. Indeed, IIEF-5 score (OR = 0.87, 95 %CI: 0.81–0.93, *P* < 0.001), primary infertility (OR = 0.55, 95 %CI: 0.31–0.99, *P* = 0.044), ART semen collection (OR = 3.25, 95 %CI: 1.84–5.73, *P* < 0.001), female factor (OR = 0.16, 95 %CI: 0.06–0.42, *P* < 0.001), both male and female factor infertility (OR = 0.17, 95 %CI: 0.09–0.33, *P* < 0.001), and unknown cause of infertility (OR = 0.26, 95 %CI: 0.12–0.59, *P* = 0.001) were independently associated with anxiety (Table 3).

**Table 3:** Multivariable logistic regression analysis of ED, depression, and anxiety risk factors

Variables	OR	95 % CI	P
**Erectile dysfunction**			
Marriage duration (years)	1.088	1.014–1.168	0.019
Intercourse frequency per month	0.744	0.639–0.867	< 0.001
TOIF	7.247	3.872–13.564	< 0.001
IELT (min)	0.797	0.715–0.887	< 0.001
**Depression**			
Sexual desire			
Normal	Reference		
General	0.954	0.391–2.328	0.917
Low	3.514	1.107–8.987	0.031
Sexual satisfaction score	0.605	0.456–0.803	0.001
ART semen collection	3.391	1.686–6.818	< 0.001
Cause of infertility			
Male factors	Reference		
Female factors	0.000	0	0.997
Both factors	0.373	0.159–0.887	0.024
Unknown	0.124	0.016–0.985	0.048
**Anxiety**			
IIEF-5 score	0.870	0.812–0.931	< 0.001
Primary infertility	1.812	1.015–3.236	0.044
ART semen collection	3.247	1.839–5.734	< 0.001
Cause of infertility			
Male factors	Reference		
Female factors	0.162	0.063–0.418	< 0.001
Both factors	0.169	0.087–0.327	< 0.001
Unknown	0.260	0.115–0.589	0.001

*OR* odds ratio, *CI* confidence interval, *IIEF-5* simplified International Index of Erectile Function, *TOIF* timely ovulation intercourse failure, *IELT* intra-vaginal ejaculation latency time, *ART* assisted reproduction technology

Table 3 also shows that other factors are independently associated with ED, psychological disorders, and sexual performance. Among all patients, marriage duration (OR = 1.09, 95 %CI: 1.01–1.17, *P* = 0.019), intercourse frequency (PR = 0.74, 95 %CI: 0.64–0.87, *P* < 0.001), TOIF (OR = 7.25, 95 %CI: 3.87–13.56, *P* < 0.001), and IELT (OR = 0.80, 95 %CI: 0.72–0.89, *P* < 0.001) were independently associated with ED (Table 3). Low libido (OR = 3.51, 95 %CI: 1.11–8.99, *P* = 0.031), sexual satisfaction score (OR = 0.61, 95 %CI: 0.46–0.80, *P* = 0.001), assisted reproductive technology (ART) semen collection (OR = 3.39, 95 %CI: 1.69–6.82, *P* < 0.001), both male and female factor infertility (OR = 0.37, 95 %CI: 0.16–0.89, *P* = 0.024), and unknown cause of infertility (OR = 0.12, 95 %CI: 0.02–0.99, *P* = 0.048) were independently associated with depression (Table 3).

### Multivariable logistic regression analysis of ED, depression, and anxiety risk factors according to the type of infertility

Table 4 shows that the associated factors of primary and secondary infertile men are different. Indeed, TOIF was independently associated with ED in primary (OR = 6.09, 95 %CI: 2.55–14.52, *P* < 0.001) and secondary infertility (OR = 9.23, 95 %CI: 2.73–31.20, *P* < 0.001). The sexual satisfaction scores were independently associated with ED in primary (OR = 0.33, 95 %CI: 0.23–0.47, *P* < 0.001) and secondary infertility (OR = 0.23, 95 %CI: 0.14–0.40, *P* < 0.001) (Table 4).

**Table 4:** Multivariable logistic regression analysis of ED, depression, and anxiety risk factors among primary and secondary infertile men

Variable	Primary infertile men			Secondary infertile men		
	OR	95%CI	P	OR	95%CI	P
**Erectile dysfunction**						
TOIF	6.091	2.549-14.522	<0.001	9.226	2.728-31.199	<0.001
Sexual satisfaction score	0.326	0.226-0.470	<0.001	0.232	0.135-0.398	<0.001
**Depression**						
Sexual desire						
Normal	Reference					
General	0.813	0.280-2.355	0.702	-	-	-
Low	5.501	1.340-22.693	0.018	-	-	-
Sexual satisfaction score	0.570	0.397-0.819	0.002	-	-	-
ART semen collection	4.292	1.720-10.711	0.002	3.621	1.115-11.763	0.032
Cause of infertility						
Male factors	Reference			-	-	-
Female factors	0	0	0.998	-	-	-
Both factors	0.304	0.086-1.073	0.064	-	-	-
Unknown	0.101	0.011-0.898	0.040	-	-	-
IIEF-5 score	-	-	-	0.821	0.732-0.922	0.001
**Anxiety**						
BMI	-	-	-	0.776	0.654-0.921	0.004
TOIF	3.998	1.796-8.897	0.001	-	-	-
ART semen collection	3.437	1.647-7.173	0.001	3.255	1.095-9.672	0.034
Cause of infertility						
Male factors	Reference			Reference		
Female factors	0.196	0.067-0.571	0.003	0.117	0.011-1.212	0.072
Both factors	0.097	0.036-0.259	<0.001	0.171	0.054-0.544	0.003
Unknown	0.283	0.116-0.693	0.006	0.083	0.006-1.133	0.062
IIEF-5 score	-	-	-	0.716	0.588-0.870	0.001
IELT (min)	-	-	-	0.726	0.556-0.948	0.019
Sexual satisfaction score	-	-	-	2.861	1.435-5.705	0.003

*OR* odds ratio, *CI* confidence interval, *IIEF-5* simplified International Index of Erectile Function, *TOIF* timely ovulation intercourse failure, *IELT* intra-vaginal ejaculation latency time, *ART* assisted reproduction technology

Low libido (OR = 5.50, 95 %CI: 1.34–22.69, *P* = 0.018), sexual satisfaction scores (OR = 0.57, 95 %CI: 0.40–0.82, *P* = 0.002), and unknown cause of infertility (OR = 0.10, 95 %CI: 0.01–0.90, *P* = 0.040) were independently associated with depression in primary infertility, but not in secondary infertility. ART semen collection was independently associated with depression both in primary (OR = 4.29, 95 %CI: 1.72–10.71, *P* = 0.002) and secondary (OR = 3.62, 95 %CI: 1.12–11.76, *P* = 0.032) infertility. IIEF-5 scores were associated with depression in secondary infertility (OR = 0.82, 95 %CI: 0.73–0.92, *P* = 0.001) (Table 4).

TOIF (OR = 4.00, 95 %CI: 1.80–8.90, *P* = 0.001), female factor infertility (OR = 0.20, 95 %CI: 0.07–0.57, *P* = 0.0.003), and unknown factor infertility (OR = 0.28, 95 %CI: 0.12–0.69, *P* = 0.006) were independently associated with anxiety in primary infertility. BMI (OR = 0.78, 95 %CI: 0.65–0.92, *P* = 0.004), IIEF-5 scores (OR = 0.72, 95 %CI: 0.59–0.87, *P* = 0.001), IELT (OR = 0.73, 95 %CI: 0.56–0.95, *P* = 0.019), and sexual satisfaction scores (OR = 2.86, 95 %CI: 1.44–5.71, *P* = 0.003) were independently associated with anxiety in secondary infertility. Finally, ART semen collection (primary: OR = 3.44, 95 %CI: 1.65–7.17, *P* = 0.001; secondary: OR = 3.26, 95 %CI: 1.10–9.67, *P* = 0.034) and both male and female factor infertility (primary: OR = 0.10, 95 %CI: 0.04–0.26, *P* < 0.001; secondary: OR = 0.17, 95 %CI: 0.05–0.54, *P* = 0.003) were associated with the two types of infertility (Table 4).

## Discussion

Secondary infertility is a particular type of infertility and mainly refers to couples that have offspring but cannot successfully conceive again. In China, there are many couples with secondary infertility, and the number has surged in recent years due to the change in the family planning policies. Although these couples have a strong wish for pregnancy, most of them are older than the optimal reproductive age, with difficulties in conceiving naturally [28, 29].

The purpose of this study was to determine whether primary or secondary infertility is associated with ED, psychological disorders, and sexual performance, and to investigate whether the associated factor of ED, psychological disorders, and sexual performance are different between men with primary vs. secondary infertility. The results suggest that the frequency of ED in men of Chinese couples with secondary infertility was higher than that of men in the primary infertility group (46.5 % vs. 26.7 %), and the quality of sexual life was lower. Some overlap is observed in factors associated with ED, psychological disorders, and sexual performance between primary and secondary infertility, but some factors are distinct.

A previous study showed that the frequency of sexual life during pregnancy attempts is increased compared to usual, and the median monthly intercourse frequency is 7 [30], which seems to require male partners to maintain better sexual function to satisfy the couple’s sexual life. On the other hand, the present study showed that the male partners in the secondary infertility group showed worse sexual function and significantly lower quality of sexual life compared with the males in the primary infertility group. About 17.2 % of the men had a long period of low sexual desire, the median monthly sexual frequency was 4.5, and 46.5 % vs. 26.7 % of the male partners with secondary and primary infertility, respectively, reported ED. Although the incidence of ED was lower than the 57.8 % observed in another Chinese study [31], this frequency was still higher than 40.6 % among men aged > 40 years in China [32]. In addition to factors such as age, marriage duration, and couple’s relationship, it should be considered that unresolved infertility might play a role in worsening ED. Therefore, male partners in secondary infertile couples have low levels of overall sexual satisfaction. Although the multivariable analysis indicated that the factors affecting ED were the same between primary and secondary infertility, the magnitude of their contribution was different.

TOIF frequently occurs in couples with childbearing needs. It is defined as a failure of ovulation and intercourse coordination, which is a type of situational ED. The occurrence of TOIF is often related to low libido caused by forced sexual intercourse, and it affects the chance of natural conception. Therefore, male reproductive function and sexual function need to be considered as a whole [30]. In the present study, TOIF was found in the two groups of participants, and both groups showed high awareness rates of ovulation of their female partners, which was higher than in a previous study [31]. Nevertheless, the frequency of TOIF in the secondary infertility group was significantly higher than that in the primary infertility group, which indicates that clinically, couples with secondary infertility should pay special attention to their intercourse efficiency. Of course, the frequency of intercourse failure in males with ED significantly increased during ovulation. This situation was observed in both the secondary and primary infertility groups, indicating that if there is a previous history of ED, it might be necessary to consider giving erectile dysfunction drugs that could increase the success rate of timely sexual intercourse during ovulation.

The occurrence of ED is often affected by a variety of factors, mainly divided into psychological, organic, and mixed types [8, 33, 34]. With the increase of age, the erectile function of men will gradually decline, and the degree of depression is more likely to affect the patient’s sexual desire and affect the erectile function [8, 33, 34]. In this study, sexual satisfaction in general was associated with ED in men with secondary infertility, suggesting that older couples preparing for pregnancy should improve the quality of their sexual life from both emotional and physiological aspects, and the low frequency of intercourse can also predict the status of ED. It is worth noting that low sexual satisfaction was also an independent risk factor for the occurrence of ED in male partners of couples with primary infertility. We believe that this is related to the fact that young men are more dependent on high expectations to induce erections. This is supported by the subgroup analysis that showed differences in ED and sexual satisfaction between younger and older men. The degree of sexual desire in patients with ED in the primary infertility group was not different from that of the secondary infertility group. The TOIF frequency of nearly 20 % of men in the primary infertility group could explain this problem.

Andrological diseases are mostly caused by physical and mental disorders [8, 33, 34]. Many patients with andrological diseases have different degrees of mental and psychological problems. The incidence of depression and anxiety disorders in patients with andrological diseases is significantly higher than that in the general population [17, 35, 36]. Psychological factors are often important factors influencing disease outcomes and communication between physicians and patients, especially for patients with andrological diseases [37]. The long time to prepare for pregnancy and the uncertainty of the fertility outcome increase the stress of the husband and wife. This increasing pressure may not only bring negative emotions but may also affect male sexual and reproductive functions. A study analyzed the effects of psychological stress on male hormones and sperm quality of male partners, noting that psychological stress first reduces serum total testosterone levels and secondly increases serum luteinizing hormone and follicle-stimulating hormone levels, suggesting that stress management might be required to improve male fertility [38]. We included only male partners from infertile couples in our study and found that the incidence of depression in the men in the primary and secondary infertility groups was 15.5 % and 14.0 %, respectively, while the incidence of anxiety was 38.4 % and 27.9 %, without significant difference in the frequency of anxiety, but with a significantly higher GAD-7 anxiety scale score in primary infertility, indicating that men who are preparing for pregnancy for the first time are more likely to have anxiety in the face of unpredictable fertility outcomes.

In this study, the multivariable logistic regression analyses showed that poor erectile function was independently associated with marriage duration, intercourse frequency, TOIF, and IELT. The higher TOIF frequency might explain why the prevalence of ED is higher in secondary infertile men. Furthermore, sexual desire, sexual satisfaction, ART semen collection, and cause of infertility were independently associated with depression, and IIEF-5 score, secondary infertility, ART semen collection, and cause of infertility were independently associated with anxiety. For men in the primary infertility group, TOIF and sexual desire were independently associated with ED, sexual desire, sexual satisfaction, ART semen collection, and cause of infertility were independently associated with depression, and TOIF, ART semen collection, and cause of infertility were independently associated with anxiety. In secondary infertility, TOIF and sexual satisfaction were independently associated with ED, ART semen collection, and IIED-5 scores were independently associated with depression, and body mass index (BMI), ART semen collection, cause of infertility, IIEF-5 score, IELT, and sexual satisfaction were independently associated with anxiety. Although TOIF appears less frequently than men in the secondary infertility group, the psychological effects of TOIF appear to be greater in men in the primary infertility group. Chinese men with infertility are prone to psychological disorders [5, 6], and this should be considered in the management of such patients.

This study has limitations. First, other scales of andrological issues (such as International-Prostatic Symptom Score and the National Institutes of Health Chronic Prostatitis Symptom Index) were not used, thus failing to refine the factors that could be associated with ED. Second, the study population was from specialized clinics and might not represent all men with infertility issues. In addition, the sample size of the present cross-sectional study was small. Third, the difference in age between the two fertility types is a confounding factor. Therefore, we conducted multivariable analyses and subgroup analyses. In the multivariable analysis, we found that primary infertility was an independent risk factor for anxiety. The desire and pursuit of an offspring might cause anxiety in the primary infertile population, while secondary infertile patients might have less pressure on bearing a child since they already have a child. This is supported by the literature [39]. Nevertheless, the results could help the clinicians quickly and effectively identifying patients with ED, depression, and anxiety and propose management methods. In addition, it suggests that clinicians should pay close attention to the role of psychological factors when making a diagnosis or treatment in those patients. Of course, the study should also include female partners’ sexual function and psychological status, and evaluate male and female factors together, which will be supplemented in our subsequent studies.

## Conclusions

This study provides evidence that although primary vs. secondary infertility was not independently associated with ED, the prevalence of ED in male partners of couples with secondary infertility in China was higher than that of the males in the primary infertility group and that they had decreased sexual quality of life. TOIF occurs more frequently in men with secondary infertility and might be one of the factors for male fertility disorders. Moreover, primary infertility is an independent risk factor for anxiety. The study suggests that when a male partner of a secondary infertile couple seeks medical help, clinicians should pay attention to combining sexual dysfunction and psychological treatment based on reproductive abnormalities.

## Data Availability

The datasets used and/or analyzed during the current study are available from the corresponding author on reasonable request.

## Abbreviations

ED: Erectile dysfunction
IIEF-5: International Index of Erectile Function
PHQ-9: Patient Health Questionnaire-9
GAD-7: 7-item Generalized Anxiety Disorder Scale
TOIF: Timely ovulation intercourse failure
WHO: World Health Organization
ORs: Odds ratios
CIs: Confidence intervals
